# D-dimer as a biomarker for assessment of COVID-19 prognosis: D-dimer levels on admission and its role in predicting disease outcome in hospitalized patients with COVID-19

**DOI:** 10.1371/journal.pone.0256744

**Published:** 2021-08-26

**Authors:** Ayusha Poudel, Yashasa Poudel, Anurag Adhikari, Barun Babu Aryal, Debika Dangol, Tamanna Bajracharya, Anil Maharjan, Rakshya Gautam

**Affiliations:** 1 Nepal Korea Municipality Friendship Hospital, Thimi, Bhaktapur, Nepal; 2 B & B Hospital, Gwarko, Lalitpur, Nepal; 3 B. P. Smriti Community Hospital, Basundhara, Kathmandu, Nepal; 4 Vayodha Hospitals, Balkhu, Kathmandu, Nepal; 5 KIST Medical College and Teaching Hospital, Imadol, Lalitpur, Nepal; 6 Alka Hospital, Jawalakhel, Lalitpur, Nepal; Ohio State University, UNITED STATES

## Abstract

**Introduction:**

Coronavirus Disease 2019 is a primarily respiratory illness that can cause thrombotic disorders. Elevation of D-dimer is a potential biomarker for poor prognosis in COVID-19, though optimal cutoff value for D-dimer to predict mortality has not yet been established. This study aims to assess the accuracy of admission D-dimer in the prognosis of COVID-19 and to establish the optimal cutoff D-dimer value to predict hospital mortality.

**Methods:**

Clinical and laboratory parameters and outcomes of confirmed COVID-19 cases admitted to four hospitals in Kathmandu were retrospectively analyzed. Admitted COVID-19 cases with recorded D-dimer and definitive outcomes were included consecutively. D-dimer was measured using immunofluorescence assay and reported in Fibrinogen Equivalent Unit (μg/ml). The receiver operating characteristic curve was used to determine the accuracy of D-dimer in predicting mortality, and to calculate the optimal cutoff value, based on which patients were divided into two groups and predictive value of D-dimer for mortality was measured.

**Results:**

182 patients were included in the study out of which 34(18.7%) died during the hospital stay. The mean admission D-dimer among surviving patients was 1.067 μg/ml (±1.705 μg/ml), whereas that among patients who died was 3.208 μg/ml (±2.613 μg/ml). ROC curve for D-dimer and mortality gave an area under the curve of 0.807 (95% CI 0.728–0.886, p<0.001). Optimal cutoff value for D-dimer was 1.5 μg/ml (sensitivity 70.6%, specificity 78.4%). On Cox proportional hazards regression analysis, the unadjusted hazard ratio for high D-dimer was 6.809 (95% CI 3.249–14.268, p<0.001), and 5.862 (95% CI 2.751–12.489, p<0.001) when adjusted for age.

**Conclusion:**

D-dimer value on admission is an accurate biomarker for predicting mortality in patients with COVID-19. 1.5 μg/ml is the optimal cutoff value of admission D-dimer for predicting mortality in COVID-19 patients.

## Introduction

Coronavirus disease 2019 (COVID-19), caused by the Severe acute respiratory syndrome coronavirus 2, was first recorded in Wuhan, the capital of Hubei province of China in December 2019 [1]. While COVID-19 is primarily a respiratory illness, it can affect multiple organ systems including gastrointestinal, hepatic, cardiac, neurological, and renal systems [1–3]. Thrombotic complications and coagulopathies including Disseminated intravascular coagulopathy are common in COVID-19, likely reflecting activation of the coagulation cascade due to viremia or cytokine storm, or possibly due to superinfection and organ dysfunction [4].

D-dimer is a fibrin degradation product, widely used as a biomarker for thrombotic disorders. A D-dimer value less than 0.5 μg/mL is usually considered normal, and values increase with increasing age and in pregnancy. The level of D-dimer rises with increased severity of community-acquired pneumonia [5]. Following the outbreak of the COVID-19 pandemic, D-dimer has been identified as a potential indicator for its prognosis in COVID-19 patients. Admission day D-dimer has shown promise for predicting the disease severity in multiple studies [6–9].

Accurate and widely available prognostic biomarkers can be very useful in the management of COVID-19. This multi-center study aims to assess elevation in D-dimer at the time of admission as a possible prognostic indicator of mortality in COVID-19 patients. The cutoff value used for D-dimer shows significant variation between the published studies, and there seems to be no consensus yet on what the best cutoff value is to predict severity or mortality. We thus aim to establish the optimal cutoff value for D-dimer that can be used clinically for predicting mortality in COVID-19 patients.

## Materials and methods

This retrospective study was conducted in four different tertiary care centers in the capital of Nepal, each of which were designated hospitals for COVID-19 patients. Ethical approval was granted by the Nepal Health Research Council Ethical Review Board. Permission and approval were taken from each hospital prior to the initiation of the study. Standards for Reporting Diagnostic accuracy studies (STARD) 2015 reporting guidelines were followed.

The sample size was calculated using the methodology described by Buderer, with an expected sensitivity of 92.3% and an expected specificity of 83.3% taken from a reference study [8, 10]. The mortality rate was estimated at 20% based on a review of hospital records. The precision and confidence interval were fixed at 10% and 95% respectively. The estimated sample size for sensitivity and specificity was calculated to be 137 and 67 respectively, and the larger value of 137 was selected.

### Patient selection

Adults (aged 18 years or older) diagnosed with COVID-19 by Reverse transcription polymerase chain reaction (RT-PCR) and admitted to one of the four study centers between March 1, 2020, and December 31, 2020 were screened for inclusion in the study. Asymptomatic cases with Peripheral oxygen saturation (SpO_2_) less than 94% and symptomatic cases were consecutively enrolled in the study. Exclusion criteria included cases without recorded D-dimer values at admission, presence of other infections, prior anticoagulant use, and deep vein thrombosis/pulmonary embolism (DVT/PE), and cases without recorded definitive outcomes (death or survival).

### Data collection

All demographic, clinical, and outcome data were extracted from the patients’ hospital record files. Demographic characteristics of patients (age, sex, and ethnicity), D-dimer on admission, SpO_2_ on admission, length of hospital stay, and outcome were recorded for each patient. All data were recorded in a standardized data collection form using standard units for measurement and verified by four physicians, one from each center. The outcome of the patient was recorded as the primary outcome (survival–discharged if PCR negative, asymptomatic, and SpO_2_≥ 94%) or non-survival (mortality due to any cause). The secondary outcome variable was the total duration of hospital stay.

### Laboratory testing

Blood samples for D-dimer assessment were collected within 24 hours of admission and sent to the respective hospital laboratories. All measurements were done in the laboratories within 2 hours of sample collection.

D-dimer was measured by immunofluorescence using kits from different manufacturers in different centers. The different analyzers used were STANDARD F200 Analyzer (SD Biosensor, Korea), POCT Axceed P200 (Bioscience (Tianjin) Diagnostic Technology Co., Ltd), Getein 1100 Immunofluorescence Quantitative Analyzer (Getein Biotech Inc., China), and mispa-i2 (Agappe Diagnostics Ltd., India). All the kits used had a biological reference range of <0.5 μg/ml, and all results were reported in Fibrinogen Equivalent Units (FEU, μg/ml).

All cases received low molecular weight heparin (LMWH), steroids, and remdesivir unless contraindicated.

### Statistical analysis

Continuous variables were expressed as mean ± standard deviation. Categorical variables were presented as n (%). The difference between the two cohorts was determined using the independent two-sample T-test for continuous variables. For categorical variables, Fisher’s exact test was used when one or more cells in the contingency table had counts of less than 5. A chi-squared test was used for other categorical variables. Duration of hospital stay between groups was compared using the Mann-Whitney U test. The accuracy of D-dimer as a predictor of mortality was calculated using the receiver operator characteristics (ROC) curve. The area under the curve, sensitivity, and specificity was calculated from the ROC curve. The optimal cutoff value for D-dimer was determined by the value corresponding to the point in the curve closest to the top-left of the ROC graph. Based on this cut-off value, patients were categorized into two groups. The 30-day outcomes of these two groups were compared using Kaplan-Meier survival analysis. Cox proportional hazards regression analysis was performed with a 95% confidence interval. Univariate analysis was performed first and only variables showing significant results in univariate analysis were included in calculating the adjusted hazard ratio. The software used for statistical analysis was Statistical Package for Social Sciences (SPSS) version 26. A p-value less than 0.05 was considered statistically significant.

## Results

A total of 182 eligible candidates were enrolled in the study, among which 113 (62.1%) were male and 69 (37.9%) were female. The mean age of enrolled participants was 58.16 years (±15.65 years).

**Fig 1:**
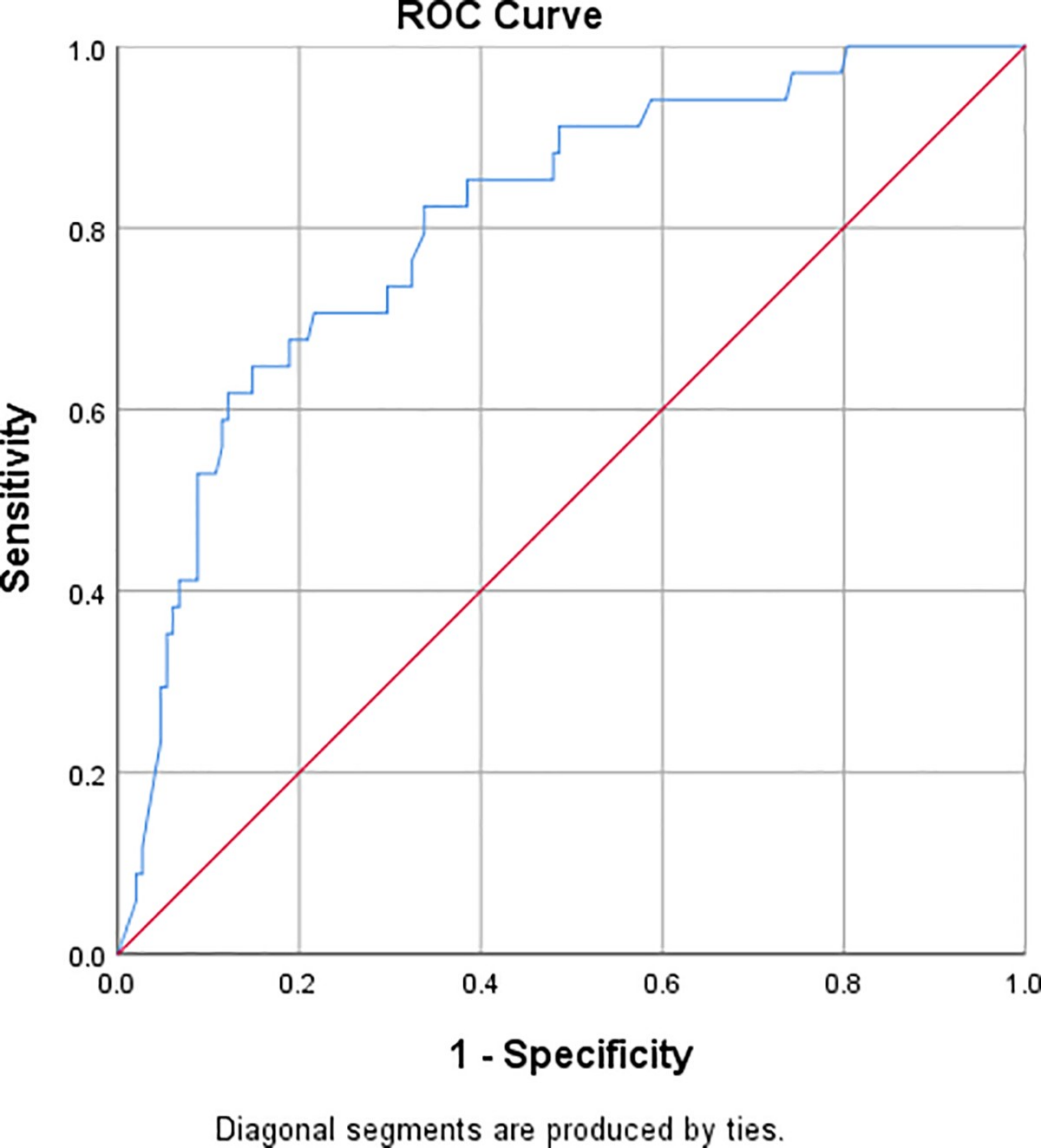
ROC curve for D-dimer as a predictor of in-hospital mortality.

34 (18.7%) patients died during hospital stay. One patient was discharged on the patient’s request and one patient left against medical advice. The area under the curve for receiver operating characteristic (ROC) curve for D-dimer values on admission against patient outcome (Fig 1) was 0.807 (95% CI 0.728–0.886, P<0.001). The optimal cutoff value of D-dimer to predict in-hospital mortality was determined by minimizing the Euclidian distance from the curve to (0,1), i.e., by measuring the shortest distance from the top-left corner of the ROC plot. The optimal cutoff value was found to be 1.5 μg/ml with a sensitivity of 70.6% and a specificity of 78.4%.

Based on the cutoff value of 1.5 μg/ml, all patients were categorized into two groups for comparison, summarized in Table 1. Among patients with admission D-dimer less than 1.5 μg/ml, 59 had underlying medical conditions (Hypertension 38, Diabetes Mellitus 32, hypothyroidism 9, COPD 3, Chronic kidney disease 1, depression 2, dyslipidemia 1, anxiety 1, coronary artery disease 1, asthma 1). Similarly, among patients with admission D-dimer more than 1.5 μg/ml, 28 patients had comorbidities (Diabetes mellitus 18, Hypertension 17, COPD 4, hypothyroidism 3, Atrial fibrillation 2, dyslipidemia 2, CKD 1, depression 1, hyperthyroidism 1). A significant difference was observed between the groups in age, whereas comorbidities did not show a statistically significant difference.

**Table 1:** Summary of 182 patients with COVID-19.

Variables	Total	D-dimer< 1.5μg/ml	D-dimer ≥ 1.5 μg/ml	p value*
		N = 126	N = 56	
Age (Mean ±SD)	58.16±15.65	55.67±15.89	63.75±13.63	0.001
Age≥60 years (n)	90	53	37	0.003
Patients with Underlying conditions (n)	87	59	28	0.692
Diabetes Mellitus	50	32	18	0.347
Hypertension	55	38	17	0.979
COPD	7	3	4	0.204
Hypothyroidism	12	9	3	0.758
Death n(%)	34 (18.7%)	10 (7.9%)	24 (42.9%)	<0.001
Discharged n(%)	148 (81.3%)	116 (92.1%)	32 (57.1%)	
D-dimer (Mean±SD)	1.48±2.08	0.44±0.36	3.83±2.42	
D-dimer (Range)	0.02->10	0.02–1.48	1.52->10	
Mean duration of stay in days (Mean±SD) ^#^	10.59±6.53	10.03±5.54	12.65±9.18	0.165

*Independent sample t-test, Chi-squared test, Fisher’s exact test, and Mann-Whitney U test were used as appropriate.

^#^Among individuals routinely discharged from the hospital (n = 146).

The mean D-dimer values on admission by age and sex are summarized in Table 2.

**Table 2:** Admission D-dimer values based on age and sex.

Variables	Mean admission D-dimer (μg/ml)
Age<60 years	1.01±1.50
Age≥60 years	1.96±2.46
Male	1.29±1.84
Female	1.79±2.40

126 out of 182 patients had D-dimer on admission less than 1.5 μg/ml, out of which 10 patients (7.9%) died during hospital stay and 116 were discharged (including one discharge on patient request). Of the 56 patients who, on admission, had D-dimer of 1.5 μg/ml and above, 24 (42.9%) died and 32 were discharged (including one against medical advice).

The mean admission D-dimer among patients who survived was 1.067 μg/ml (±1.705 μg/ml), whereas that among patients who died was 3.208 μg/ml (±2.613 μg/ml). This is a highly statistically significant difference (p<0.001, independent samples T-test).

**Fig 2:**
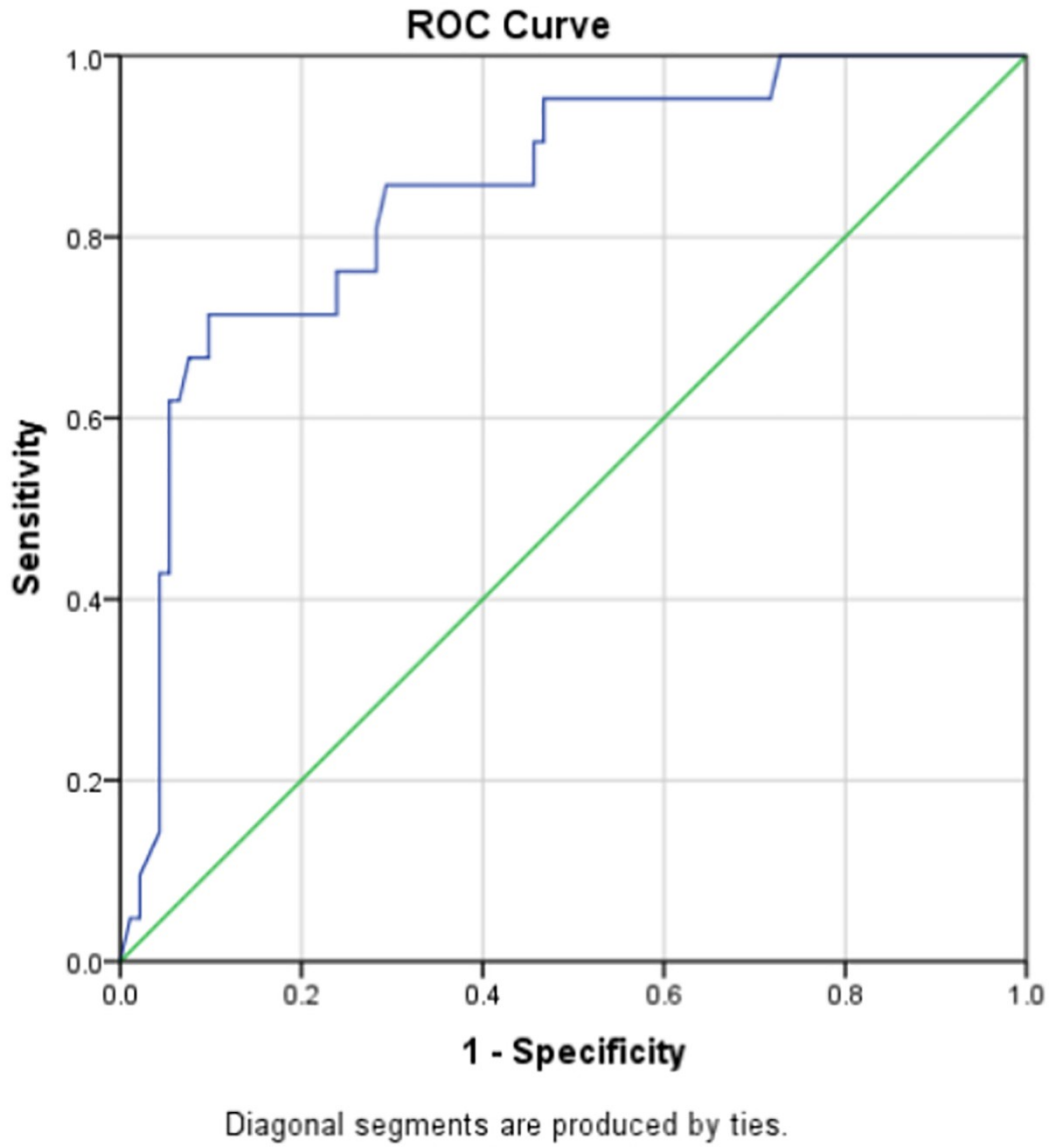
ROC curve for D-dimer as a predictor of in-hospital mortality in males.

**Fig 3:**
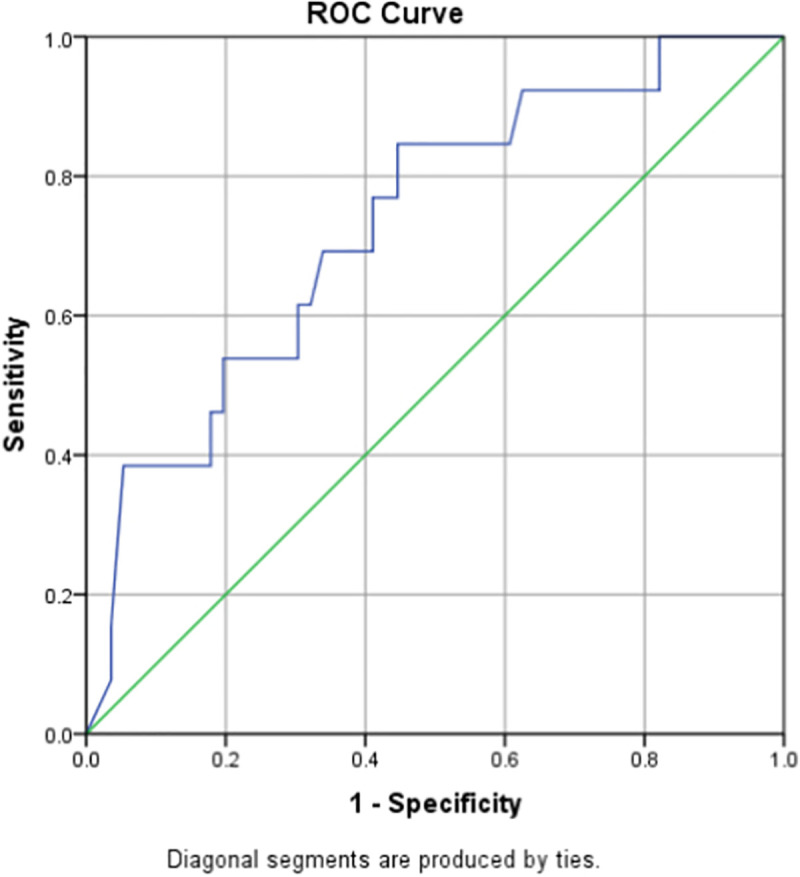
ROC curve for D-dimer as a predictor of in-hospital mortality in females.

Subgroup analysis of male and female patients showed different optimal cutoff values; 1.90 (0.759–0.94) with sensitivity of 71.4% and specificity of 90.2% for males and 1.46 (0.583–0.880) with sensitivity of 69.2% and specificity of 66.1% for females (Figs 2 and 3).

The peripheral oxygen saturation measured via pulse oximeter at the presentation to the hospital and D-dimer levels at hospital admission were analyzed for presence of any correlation. The Pearson correlation between the two was found to be -0.134 with alpha level set to 0.05 and a p-value of 0.085. This demonstrates that there is no correlation between oxygen saturation and D-dimer at admission.

Comparison of duration of hospital stay between the two groups using Mann-Whitney U test did not show a statistically significant difference (p = 0.236). Among the 146 patients who recovered and were discharged routinely (excluding discharge against medical advice), 113 had D-dimer at the time of admission of less than 1.5 μg/ml, while 31 had D-dimer on admission higher than 1.5 μg/ml. A separate analysis of these patients, who were discharged routinely, included in Table 1, again failed to show a significant difference (p = 0.165).

**Fig 4:**
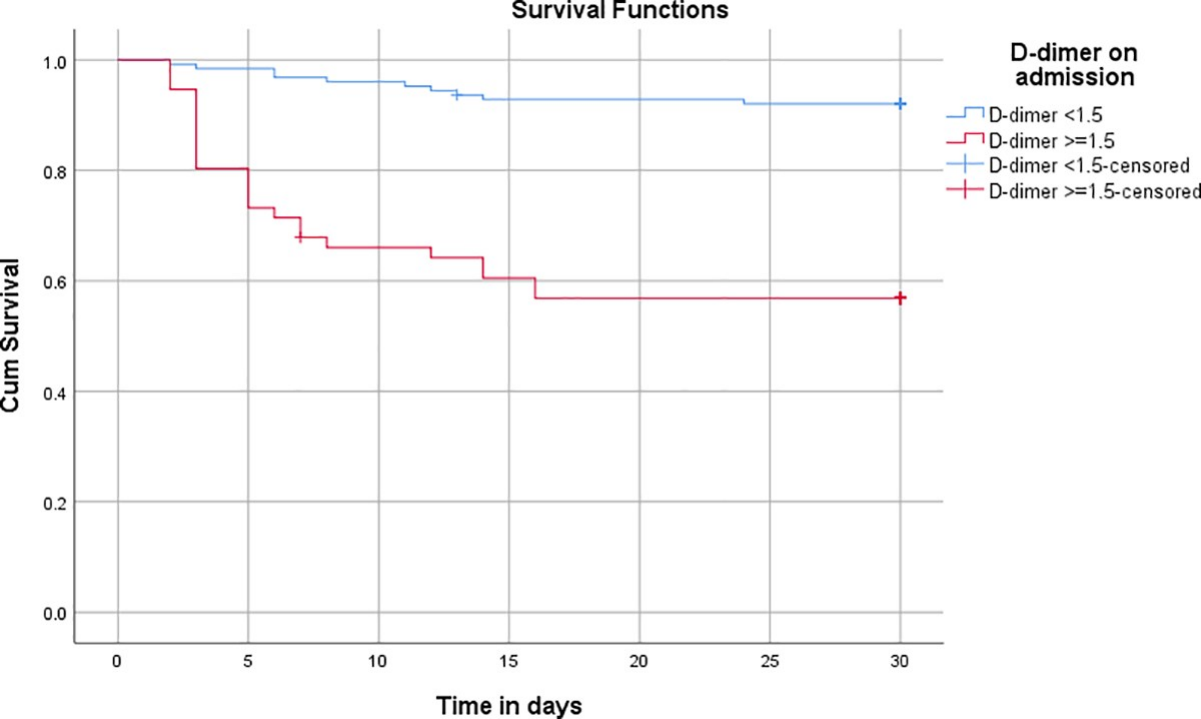
Survival analysis using the Kaplan Meier procedure.

30-day survival of patients was analyzed using the Kaplan-Meier procedure, which showed a significant difference (Log-rank (Mantel-Cox) of 0.00) in survival between the low D-dimer and high D-dimer groups at 30 days (Fig 4).

The results of univariate Cox regression, as shown in Table 3, performed using high admission D-dimer (higher than 1.5 μg/ml), age, sex, and major comorbidities (hypertension, diabetes mellitus, chronic kidney disease) are summarized in Table 3. Only admission D-dimer and age had significant hazard ratios when analyzed separately. When adjusted for age, the hazard ratio for high D-dimer was 5.862 (2.751–12.489 at 95% CI, p<0.001), whereas when adjusted for age, sex, presence of hypertension, diabetes mellitus, and chronic kidney disease, the hazard ratio for D-dimer was 6.823 (3.105–14.991 at 95% CI, p<0.001).

**Table 3:** Univariate Cox regression analysis.

Variable	Unadjusted hazard ratio	95.0% CI		Significance
		Lower	Upper	
Age	1.033	1.010	1.057	0.005*
Sex	1.003	0.502	2.003	0.994
D-dimer on admission	6.809	3.249	14.268	<0.001*
Diabetes Mellitus	0.948	0.442	2.030	0.890
Hypertension	1.280	0.633	2.586	0.492
Chronic Kidney Disease	3.200	0.437	23.447	0.252

*Significant.

Further analysis among the deceased cases was done taking into consideration the laboratory parameters including hemoglobin, WBC with lymphocyte, platelet count, C- reactive protein, prothrombin time (PT) and activated partial thromboplastin time (aPTT) as shown in Table 4. These variables have a role in determining coagulopathy in the patients. CRP and aPTT showed statistically significant elevation among deceased cases with high D- dimer values.

**Table 4:** Basic laboratory values among high and low D-dimer groups.

	Mortality Among High D-Dimer cases	Mortality Among Low D-Dimer cases	
	**Hemoglobin (10/19)**	**Hemoglobin (5/9)**	**p-value**
Mean +/- SD	12.2 +/- 1.5635	12.78 +/- 1.8939	0.538
	**WBC (15/19)**	**WBC (8/9)**	
Mean +/- SD	7290 +/- 4382.305	9950 +/- 3450.329	0.535
	**Platelets (10/19)**	**Platelets (5/9)**	
Mean +/- SD	179090 +/- 118722	241000 +/- 101126.159	0.338
	**Lymphocyte (15/19)**	**Lymphocyte (8/9)**	
Mean +/- SD	0.1448 +/- 0.6757	0.15325 +/- 0.503	0.566
	**CRP (15/19)**	**CRP (7/9)**	
Mean +/- SD	137.38 +/- 82.035	60.013 +/- 31.255	0.025*
	**PT (10/19)**	**PT (4/9)**	
Mean +/- SD	16.25 +/- 2.55	12.75 +/- 1.6623	0.626
	**aPTT (10/19)**	**aPTT (5/9)**	
Mean +/- SD	47.38 +/- 20.34	42.32 +/- 13.5717	0.028*

*Significant.

Patients received plasma therapy, non-invasive and invasive ventilation as per requirement.Similarly, the immediate cause of death was analyzed among the both high and low D- dimer groups, as shown in Table 5. All deaths among included cases occurred in the hospital and were the sequelae of COVID-19. The most common cause of death was noted to be severe hypoxia due to COVID-19 pneumonia, which led to severe bradycardia and cardiac arrest. Few cases succumbed to sepsis with multiorgan failure. However, ARDS was documented only in few cases with severe respiratory failure who developed cardiac arrest secondary to severe hypoxia.

**Table 5:** Treatment received and immediate cause of death among patients with high and low D-dimer values.

	**Mortality Among High D-Dimer**		
Total patients	24	Immediate cause of death	
Received LMWH	21	Severe respiratory failure leading to cardiac arrest	16
Mechanical ventilation	11	ARDS documented among severe respiratory failure cases	4
Non-invasive ventilation	7	Sepsis	1
Received plasma therapy	8		
	**Mortality Among Low D-Dimer**		
Total patients	10	Immediate cause of death	
Received LMWH	9	Severe respiratory failure leading to cardiac arrest	6
Mechanical ventilation	2	ARDS documented among severe respiratory failure cases	1
Non-invasive ventilation	6	Sepsis	2
Received plasma therapy	4	Multiorgan failure	1

## Discussion

The current study included 182 cases admitted to the hospital with a diagnosis of COVID-19 from March to December 2020, which was in the relatively early phase of the pandemic in Nepal. Cases were managed in the hospital-based on available resources and guidelines, the latter of which were not established early on and evolved with time. D-dimer was usually measured on admission, and serial D-dimer measurement was not part of routine management. Treatment during the study period was largely symptomatic, consisting of antipyretics, analgesics, and supplemental oxygen when required. LMWH was given prophylactically to all patients without contraindications.

This study found that a higher D-dimer value on hospital admission was significantly associated with in-hospital mortality in patients of COVID-19. D-dimer is a fibrin degradation product and its main utility is in the diagnosis and management of thrombotic disorders. Before the 2019 COVID-19 pandemic, D-dimer was not considered a useful biomarker for bacterial or viral pneumonia despite some evidence to the contrary [5]. Since then, however, elevated D-dimer and thrombotic complications have been widely reported in COVID-19 patients. Guan et al. reported that D-dimer more than 0.5 μg/ml was found in 260 out of 560 patients (46%) [3]. Several studies have been conducted to study the association between initial D-dimer measurements and disease severity and outcome. A study done by Zhang et. al. in China including 343 patients concluded that D-dimer could be an early useful marker for predicting in-hospital mortality in patients. They found the optimal cutoff point for D-dimer to be 2 μg/ml [8]. Another study in China found that D-dimer value at the time of admission of more than 2 μg/ml was associated with increased odds of mortality (Odds Ratio 10.17 (95% CI 1.10–94.38) [7]. A similar study in India found the optimal cutoff value for admission D-dimer to predict hospital mortality to be 1.44 μg/ml, whereas the optimal value for highest D-dimer measurement during hospital stay for predicting hospital mortality was 2.01 μg/ml [9]. A systematic analysis published in August 2020 found that COVID-19 patients presenting with high D-dimer values were at increased risk of severe disease and mortality, and noted that no consistent cutoff value had been defined to predict adverse events [11]. A retrospective study conducted in the United States including 1065 hospitalized patients found that every 1 μg/ml increase in admission D-dimer was associated with a hazard ratio of 1.06 (95% CI 1.04–1.08, p<0.001) for all-cause mortality. However, they found D-dimer to be a poor prognostic test for predicting mortality, with an area under the curve of ROC curves for D-dimer trend to be just 0.678 [12].

A systematic review by Rostami et al. reported the mean D-dimer level to be 0.58 μg/ml in 1551 patients with mild disease and 3.55 μg/ml in 708 patients with severe disease [13]. A meta-analysis done by Gungor et al. showed that patients with elevated D-dimer on admission had a higher risk of mortality (relative risk, RR 1.82) and disease severity (RR 1.58) compared to the patients with normal levels of D-dimer [14]. A similar meta-analysis found a relative risk of mortality of 4.60 (95% CI 2.72–7.79) taking 0.5 μg/ml as the cutoff value [15]. Another meta-analysis including 6 studies found that COVID-19 patients with elevated D-dimers have worse clinical outcomes including all-cause mortality, ICU admission, and acute respiratory distress syndrome (ARDS) [16].

The area under the curve (AUC) of the ROC curve for D-dimer on admission in our study was 0.807. An area under the curve of more than 0.8 is generally considered to indicate ‘good accuracy’ of the test, whereas values above 0.7 are considered to indicate ‘fair accuracy’. This finding is in accordance with most of the published research on the topic. Zhang et al found an AUC of 0.89 in their study, whereas Oualim et al reported an AUC of 0.775 and Peiro et al. reported an AUC of 0.756 [8, 17, 18]. Our AUC value is higher than some reported values of AUC, including Naymagon et al. who showed an AUC of 0.694, Soni et al., who showed an AUC of 0.683, and He et al. who reported an AUC of 0.661 [9, 12, 19].

The optimal cutoff value for admission D-dimer for predicting mortality has not been agreed upon in the current literature. Some studies have identified optimal cutoffs based on ROC curves, but the values range anywhere from 0.67 to 2.025 μg/ml, with large variation in sensitivity and specificity as well [8, 9, 12, 17–19]. A French multicenter study published in May 2021 identified the cutoff value for admission D-dimer as 1.113 μg/ml. In our study, the optimal cutoff value was calculated based on the distance of each point in the ROC curve to the top left of the graph. This provides a good balance of sensitivity and specificity. In our study, the optimal value was identified as 1.5 μg/ml, with a sensitivity of 70.6% and a specificity of 78.4%. This cutoff represents a three-fold increase in D-dimer value from the commonly used upper limit value of 0.5 μg/ml as normal.

A recent study found that the trajectory of lab values including D-dimer in hospitalized patients had good accuracy in predicting mortality and severity of COVID-19 [20]. Considering the entire trajectory of D-dimer during hospital admission could offer better prognostic value than admission D-dimer alone, and more studies are needed in this field.

There is significant heterogeneity among studies on D-dimer and COVID-19. Different laboratories use different kits for measurement and the accuracy and reliability of measurement can vary according to the kit manufacturer. Furthermore, there is variation in reporting units. Favaloro and Thachil analyzed 20 papers on COVID-19 and D-dimer and found that most papers did not report which manufacturer and reagent kit was used and whether D-dimer values were reported in D-dimer units (DDU) or Fibrinogen equivalent units (FEU). They also found that nearly half the studies did not report normal cutoff values [21]. This lack of standardization leads to chances of pitfalls in the analysis and interpretation of D-dimer values in COVID-19.

The most common reason cited in the literature for the elevation of D-dimer includes viremia and the cytokine storm syndrome, in which the rise in pro-inflammatory cytokines (IL-2, IL-6, IL-8, IL-17, TNF-α) are inadequately controlled by the anti-inflammatory factors which overwhelm the coagulation cascade [4]. Hypoxia itself leads to activation of hypoxia-inducible transcription factor-dependent signaling pathway, predisposing to thrombosis. The disease most commonly affects elderly and comorbid patients. Advancing age and common comorbidities such as hypertension, diabetes mellitus, and cardiovascular diseases can predispose the patients to thrombosis.

A major limitation of our study is selection bias because of its retrospective nature. Only patients admitted to the hospital were included, which meant that asymptomatic patients with high oxygen saturation, who were not admitted according to hospital guidelines, were not included in the study. Some otherwise eligible cases also had to be excluded due to incomplete laboratory tests and medical records, specifically D-dimer on admission. Time from illness onset to hospital presentation may affect the D-dimer values. Since the study was conducted in four centers with their own laboratories, different kits were used for the measurement of D-dimer in different centers, which causes the potential for measurement bias due to the use of different equipment. The reference ranges of D-dimer and the units of reporting are the same for all four centers, which should mitigate this to some extent.

D-dimer is a widely available, relatively inexpensive, and easy to perform laboratory test, and our study found that it has good accuracy in predicting in-hospital mortality in COVID-19 patients. It can be used as a metric for identifying high-risk cases and can assist in choosing appropriate management. Incorporation of D-dimer into routine investigation and risk assessment of COVID-19 patients can prove useful in tackling this global health challenge.

## Conclusion

D-dimer value on admission is an accurate biomarker for predicting mortality in patients with COVID-19 and 1.5 μg/ml is the optimal cutoff value of admission D-dimer for predicting mortality in COVID-19 patients, with good sensitivity and specificity. D-dimer can thus be an easy to perform and inexpensive laboratory indicator for COVID-19 prognosis.

## Supporting information

S1 File Demographic parameters and laboratory results of patients included in the study. (SAV)

S2 File Demographic parameters, laboratory results, co-morbidities and treatment received segregated by outcome of patient. (XLSX)

S3 File Demographic parameters, D-dimer levels, oxygen requirement and co-morbidities segregated with D-dimer cut-off of 1.5. (XLSX)
